# Impact of a Prepharmacy Program on Students’ Self-Awareness of Pharmacist Professional Identity: Comparison between Virtual and In-Person Settings

**DOI:** 10.3390/pharmacy10020044

**Published:** 2022-04-09

**Authors:** Ashim Malhotra, Eugene Kreys, Xiaodong Feng

**Affiliations:** 1 Department of Pharmaceutical and Biomedical Sciences, College of Pharmacy, California Northstate University, Elk Grove, CA 95757, USA; 2 Department of Clinical and Administrative Sciences, College of Pharmacy, California Northstate University, Elk Grove, CA 95757, USA; ekreys@cnsu.edu (E.K.); xfeng@cnsu.edu (X.F.)

**Keywords:** professional identity, professional identity formation, prepharmacy, pre-matriculation program, face-to-face versus online

## Abstract

Ensuring an adequate preparation for undergraduate students transitioning to pharmacy school is challenging. A significant barrier is changing from a subordinate to a critical thinking mindset while self-identifying as a professional. Here, we aimed to (1) determine whether our prepharmacy program called “*Pr*ofessional *I*dentity and *Me*” (PRIME) could enhance learners’ self-awareness of their professional identity and (2) compare the effectiveness of the in-person and online versions of PRIME. PRIME introduced prepharmacy students to aspects of pharmacists’ professional identity including community, hospital, and interprofessional work, as well as mental health, wellness, and time and stress management skills, Top-200 drugs, prerequisite foundational sciences, and calculations. Concepts of professionalism, graduate writing, and ownership were also presented. Bridging exercises were introduced to exemplify application. We used a mixed-methods approach to assess the outcomes. The average performance in knowledge-based assessments increased before and after the PRIME program from 53.8 to 74.6% and from 47.7 to 75.9%, while the difference in the test scores was statistically significant, with a 21% increase (*p* < 0.001, 95% CI 15–26%) and a 28% improvement (*p* < 0.001, 95% CI 23–34%) for face-to-face versus virtual PRIME. The results of a student perception survey revealed PRIME was equally effective as a virtual program during the COVID-19 pandemic, suggesting transferability to other pharmacy programs.

## 1. Introduction

Professional identity (PI) refers to the adoption and internalization of a set of norms that make an individual “think, feel, and act” as a member of a community [1]. Professional identity formation (PIF) is defined as the process that leads a community of closely aligned professionals to identify, develop, and adopt a common set of characteristics, attributes, attitudes, behaviors, and beliefs associated with the efficacious discharge of their duties and daily operations [2]. PI is formed through the “persistent, sustaining social network of individuals who share and develop an overlapping knowledge base, set of beliefs, values, history, and experiences focused on a common practice” [3].

In the United States, there has recently been much discussion regarding the PI of pharmacists and pharmacy students. Pharmacists practice in a variety of fields—including community, hospital, research, education-related faculty positions—and as merchandisers and drug experts. As the practice and the roles of pharmacists have expanded in the U.S.A. and the roles of pharmacists have expanded in interprofessional health care teams, calls to define the PI of pharmacists have been taken up officially by the American Association of Colleges of Pharmacy (AACP). For example, AACP charged the Student Affairs Standing Committee to define and adopt a unified pharmacist’s PI and to encourage schools and colleges of pharmacy to “advance education aimed at the intentional formation of professional identity” [4]. However, pharmacists’ PI is challenging to define. Equally challenging has been the creation, implementation, and assessment of educational programs aimed at PIF for pharmacy students. 

Several factors impede the creation, implementation, and assessment of PIF in pharmacy programs. “Identity dissonance” between the ideal pharmacy practice environment that pharmacy students experience during schooling and the real world [5,6] is an example of an important impedance factor. Early career pharmacy-related work experience, especially that occurring before starting a pharmacy education program, positively impacts and influences pharmacy students’ PIF [7], closing this identity dissonance gap. However, there is a lack of PIF-focused pre-matriculation programs. Boolean searches conducted by the authors in PubMed, Medline, Google Scholar, and Web of Science for the terms “pre-matriculation programs” and “professional identity formation for pharmacists” did not yield much information. 

Furthermore, the COVID-19 pandemic has disrupted education on a global scale. COVID-related disruptions further complicated the creation and implementation of readiness programs that encourage the professional identity formation of first-year pharmacy students. For instance, while COVID-19 expanded the technology dependency for the delivery and assessment of the curriculum, such technology was not available everywhere, highlighting problems with student readiness and preparation for entry into pharmacy programs [8]. Importantly, the impact of this technology revolution on guidance and assistance programs such as those associated with admissions and pre-matriculation remains unassessed. 

Much effort was expended across health professions education programs to continue high-quality interprofessional education and practice (IPEP) training through the application of Meaningful Discourse and the Community of Inquiry principles [9]. As the pandemic raged, a common strategy emerged, with most interprofessional education switching to some form of online teaching [10], and many interprofessional practice programs adopting some form of telehealth-related practice [11]. However, the already challenging field of introducing IPEP foundations to first-year and even pre-matriculated students was scantily addressed. Interprofessional teamwork is a quintessential component of pharmacist professional identity and responsibility, of which pharmacy students need to have a clear understanding. 

Medical and pharmacy schools in the United States have implemented pre-matriculation programs to address the diversity in academic preparation and readiness [12,13,14,15]. However, a review of the literature demonstrates the paucity of PIF-building pre-matriculation programs. There is also a lack of systematic assessment of the impact of converting pre-professional and pre-matriculation programs from an in-person to a virtual setting on learners’ readiness for health professions education programs. 

To close these gaps, here we report the creation and implementation of a pre-matriculation program to foster pharmacist student professional identity, christened the “*Pr*ofessional *I*dentity and *Me*” (PRIME) Program. PRIME immersed pre-matriculated students with confirmed admission to our pharmacy program in activities, lectures, role-play, and team-based learning. PRIME had the following goals: (1) to enhance the professional identity of incoming pharmacy students, (2) to help review select foundational and pharmaceutical sciences content, (3) to wean students from an undergraduate mindset and foster a growth mindset, and (4) to improve learner aptitude for pharmacy school. Additionally, we report a systematic and statistical comparative analysis of the in-person and virtual versions of PRIME. The virtual version of PRIME was necessitated by the ongoing COVID-19 pandemic. To our knowledge, this is the first comparative analysis of the impact of an in-person and a virtual professional identity formation-oriented learner-centered program. 

## 2. Materials and Methods

### 2.1. Experimental Approach

The initial pilot for PRIME was conducted for the program’s quality improvement. The subsequent collection of de-identified aggregated data and their secondary analysis was exempted from review by the California Northstate University Institutional Review Board. The current study presents a retrospective causal analysis of the learner-perceived effectiveness of the delivery of PRIME. In this study, we aimed to determine whether PRIME (1) impacted self-perceptions of PIF of pre-matriculated pharmacy students, (2) enhanced students’ recall of pre-requisite foundational sciences content, (3) enabled the learners to connect the concepts of pharmacy practice with the foundational sciences, and (4) differed substantially in student perception of its delivery and effectiveness when comparing the face-to-face (F2F) pilot conducted in 2019 and the virtual iteration conducted in 2020 during the COVID-19 pandemic. This is an important research question given the complexity of imbibing PIF, the intricacies of explaining interdisciplinary and interprofessional healthcare, the heterogeneity of the content, and the lack of F2F contact between faculty and students. 

### 2.2. Environment and Student Recruitment

The California Northstate University College of Pharmacy (CNUCOP) offers a four-year traditional Doctor of Pharmacy (PharmD) program that is fully accredited by the Accreditation Council for Pharmacy Education, with 550 full-time PharmD students enrolled as of 2022, taught by 42 full-time faculty. 

Student recruitment for the PRIME program was managed by the CNUCOP Office of Admissions and Student Affairs which registered the accepted students who completed an enrollment agreement. Recruitment generally began in March. During the time, the pandemic had already resulted in “Stay-At-Home” orders in the state of California, an invitation email and a “welcome package” were sent to these students, with three additional reminders over the next four months. The following information and instructions were included (1) a flyer announcing the PRIME program, (2) a detailed class schedule for the two-week-long PRIME program, (3) computer requirements for attending the synchronous sessions during the 2020 virtual version, (4) a YouTube video highlighting the benefits of attending PRIME, and 5) email addresses of college contacts. There was documented interest among students for the PRIME program. For example, more than half of the 2020 first-year cohort (N = 46 of 79; 58%) registered for PRIME, which was scheduled two weeks ahead of the official start of pharmacy school and the mandatory attendance “orientation session” in August 2020, which did not contain sections on professional identity or professional identity formation. Among reasons for not attending, the most commonly cited reasons for both the F2F and the virtual versions of PRIME were ongoing summer classes, prior work commitment, and ongoing translocation to the city where the college is located.

### 2.3. Virtual Platform and Integration Techniques

To enable virtual learning, University IT helped override specific technical matriculation requirements and expedited the university’s online onboarding step. This allowed pre-matriculated students electronic access to our learning management system called Canvas (Instructure, Salt Lake City, UT, USA) into which, each of the classes was built with electronically available instructor-created course content. Although Canvas has in-built virtual broadcasting features such as Canvas Conference (BigBlueButton), considering the need to pre-train the students in using this feature, it was decided to instead hold daily classes using the more publicly familiar web conferencing platform Zoom (Zoom, San Jose, CA, USA). 

### 2.4. Curricular Design

PRIME comprised eight days of live synchronous classroom engagement distributed over two weeks starting July 28th and ending on August 7th, 2020. Each synchronous learning day of PRIME was six hours long, not counting a one-hour lunch break. Table 1 outlines the topics included in PRIME. In its distance education iteration, compared to the F2F pilot, we expanded PRIME into a free, two-week-long program that included multiple elements related to pharmacists’ PIF. Since PIF also depends upon an internationalization and understanding of the scientific premise of pharmacists’ daily work [1], course work in foundational prerequisite science topics included biochemistry, medicinal chemistry, anatomy and physiology, pathophysiology, calculations, and introduction to pharmacology. 

Keeping in mind the distance education environment during the COVID-19 pandemic and aspects of PIF-related courses, we added course content in the following areas: (1) mental and emotional health and resiliency, (2) introduction to graduate writing, (3) effective teamwork strategies to prepare incoming students for working in interprofessional health care teams and the Team-Based Learning modality used in some of our didactic courses, (4) introduction to interprofessional education and practice (IPEP), including added sessions on the principles of high-fidelity simulation for learning clinical skills, (5) pharmacy career opportunities and early career planning, (6) non-traditional pharmacy careers that require interdisciplinary training such as Industry Pharmacy, and (7) the principles of management, professionalism, and leadership. Each module was developed and taught by faculty or staff with expertise in that area. For example, the Top-200 drugs module introduced the concepts of drugs used at high frequency, the general medicinal and pharmacological categories they belong to, and educational resources available at the college such as AccessPharmacy resources and flash cards for familiarizing with the content. The module was designed and taught by the same faculty member who teaches this content in the first year of our PharmD program. Thirteen faculty developed and taught over 14 topics, ensuring to cross-refer to each other’s topical coverage to horizontally integrate all the topics. 

To ensure that the pharmacy students were able to successfully intellectually integrate all the information and apply it appropriately, along with forming an understanding of the pharmacist’s PI, we developed a specific curricular design for PRIME. Each module concluded with self-directed learning “practice quizzes” that were built into Canvas. Students first answered the practice quiz individually, followed by student teams taking the same quiz again. These practice quizzes were not graded, though students were shown the correct answers after the team attempt to help them learn. There was, however, a pre-and-post activity quiz that was a graded component for the foundational sciences-based pre-requisite content. After each session, we included a tailor-made “Bridge-to-Pharmacy” PIF activity, which was a simpler version of Case Based Learning. Specifically, a real-life patient case scenario, simplified in presentation and carefully linked to the topic being taught, was presented to student teams, which were allotted 30 minutes to identify the problem and develop a “concept map” of what was covered and how it applied to the real-life scenario. 

### 2.5. Survey Administration, Data Collection, and Statistical Analysis

We used a mixed-methods approach for evaluating PRIME. Overall, science, and math GPAs and baseline demographic characteristics such as gender, race/ethnicity, and age were evaluated and compared with the 2019 F2F pilot. 

Student perceptions were gathered using a retrospective survey instrument comprising 23 questions (Table 2). We based our survey on similar validated surveys for measuring learners’ skills, attitudes, and behaviors towards professional identity found in the medical education literature [16]. While we adapted the questions to make them pertinent to our work and also added a few questions, we carefully maintained the overall fidelity of the survey. Additionally, in the absence of a standardized PI or PIF survey in pharmacy education, we loosely structured our survey questions in a way that they could be subcategorized within the themes of the 2013 Center for the Advancement Pharmacy Education (CAPE) Outcomes Domains [17]. The responses were anonymously collected through SurveyMonkey, and the survey was in the form of a 5-point Likert scale in both the control (2019 F2F) and the test group (2020 Virtual). 

A parametric independent Student’s *t*-test was used when comparing incoming GPAs, a chi-square test when comparing demographics, and a non-parametric Mann–Whitney U test when comparing the results of the perception surveys. The internal consistency of the survey questions was validated using Cronbach’s alpha analysis with an alpha value ≥0.70 deemed acceptable.

Additionally, to determine the impact of PRIME on learners’ understanding of PIF, content retention, and overall PharmD program readiness, we also conducted a pretest and posttest analysis of the 2020 PRIME cohort performance and used a paired Student’s *t*-test for statistical analysis. This test assignment was proctored during both the F2F and the virtual version of PRIME. During virtual PRIME, we employed a two-device monitoring system where students answered the test on ExamSoft (Dallas, TX, USA) while being monitored by a staff member using online video streaming on a mobile device. Prior consent was obtained from the students, and we also informed them that similar proctoring may be conducted during the virtual classes during the COVID-19 pandemic. Furthermore, analysis of covariance was used to compare the difference in posttest scores between the 2019 and the 2020 cohorts, while controlling for the pretest scores. A univariate test was used to establish the homogeneity required for the analysis of covariance. The appropriateness of the use of the specific parametric test was based upon verification of normality distribution using the Shapiro–Wilk test and equality of variances using Levene’s Test. Statistical significance was set at an alpha of 0.05. IBM SPSS^®^ version 26 was used for statistical analysis.

## 3. Results

### 3.1. Comparative Analysis of PRIME Cohorts

As shown in Table 3, we found a similar distribution of race and ethnicity in the control (F2F PRIME 2019) and test (virtual PRIME 2020) cohorts (*p* = 0.455), with Asians making up 76% (2019) and 65% (2020) of the cohorts. Furthermore, the mean student age in the control and test groups was close to 24 years (*p* = 0.419). Interestingly, however, there was a statistically significant difference in the gender composition of the two cohorts, with females making up 76% of the control group but only 57% of the test group (*p* = 0.037). The undergraduate average, overall, and science GPA of the pre-matriculated students were also comparable (*p* = 0.232 and 0.367, respectively), while the average math undergraduate GPA of 3.05 in the F2F PRIME 2019 cohort was significantly higher than the 2.77 average math GPA of the virtual PRIME 2020 cohort (*p* = 0.025). 

### 3.2. Learners’ Perception and Awareness of Their Professional Identity Formation as Captured by Post-PRIME Surveys

A review of the student perception survey results for the F2F PRIME 2019 cohort revealed generally positive results, with nearly 100% of the students selecting agreed or strongly agreed to all 23 questions of the survey, including questions focused on interaction with classmates, study strategies, effectiveness in the preparation for the pharmacy curriculum, and reflection on future careers. Cronbach’s alpha analysis of the 23 questions revealed a very high level of internal consistency, with an alpha value of 0.935.

### 3.3. Learners’ Self-Perception of Their Understanding of Different Aspects of Pharmacist PIF during PRIME

**Figure 1:**
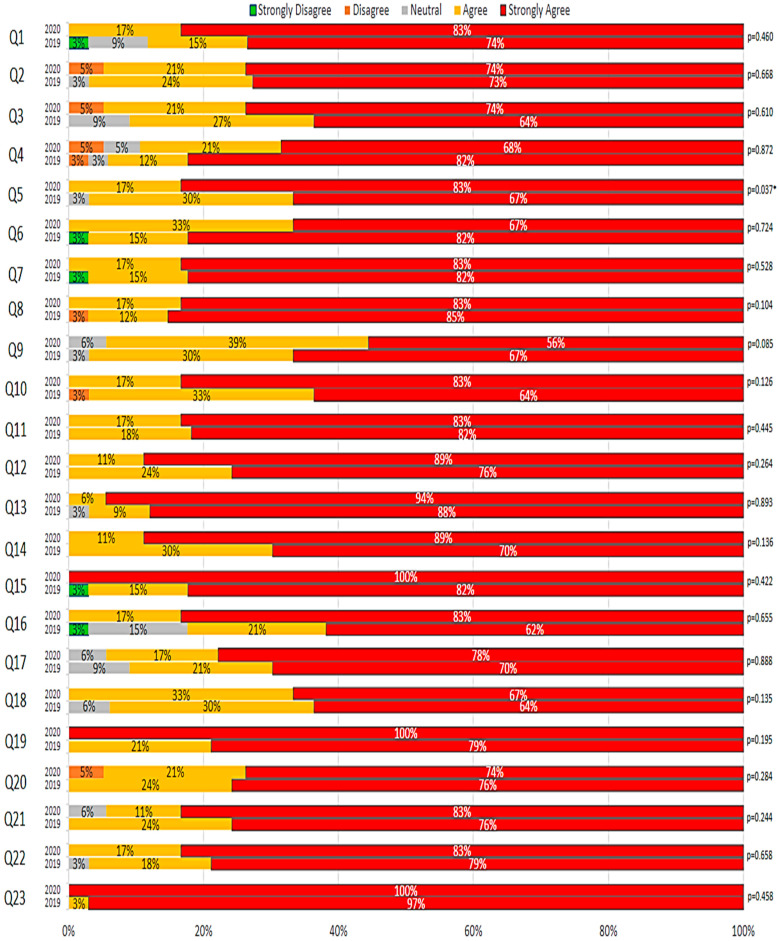
Statistical comparison of learners’ self-perception of gain in professional identity formation between F2F and virtual PRIME programs in 2019 and 2020, respectively. * indicates statistically significant difference.

As shown in Figure 1, a comparison of post-activity PRIME student perception survey results revealed very similar results between the 2019 and 2020 responses. Analysis for only a single question regarding the response to the statement “I liked the exposure I got to studying pharmacy-related subject material as an introduction to pharmacy school” resulted in a statistically significant difference (*p* = 0.037). However, this was primarily the result of the fact that 100% of the subjects in 2020 strongly agreed with the statement, while 21% of the subjects in 2019 simply agreed rather than strongly agreed.

### 3.4. Learners’ Performance on Knowledge-Based Formative Assessments 

The average performance in knowledge-based assessments increased before and after the PRIME program from 53.8 ± 22.2% to 74.6 ± 13.6% in 2019 and from 47.7 ± 21.4% to 75.9 ± 14.2% in 2020. The difference in the test scores administered before and after the PRIME program was statistically significant, with a 21% improvement in 2019 (*p* < 0.001, 95% CI 15–26%) and a 28% improvement in 2020 (*p* < 0.001, 95% CI 23–34%). A univariate test measuring the interaction between the year of the PRIME program and the pre-test scores resulted in a *p*-value of 0.40, meeting the assumption for homogeneity required for the analysis of covariance. The subsequent analysis of covariance determined that the 3.2% adjusted difference in the post-test scores between the 2019 and 2020 years of the PRIME program was not statistically significant (*p* = 0.204, 95% CI: −1.7–8.2%) when controlling for the pre-test scores. 

## 4. Discussion

### 4.1. PRIME Helps Wean Students from a “Subordinate” Mindset to Self-Identify as a Student Pharmacist

Pharmacy students must transition from a starting undergraduate mindset, characterized by sovereign and subordinate goal-completion thinking to assuming a pharmacist’s identity. Subordinate task-solving behavior is related to immediate task completion [18], avoiding an understanding or participation in relational and holistic thinking, which involves asking questions such as “How does the immediate test or classroom assignment relate to the work of a pharmacist”? Students gaining entry into the Doctor of Pharmacy programs in the United States generally have an undergraduate degree, though some may have taken pre-requisite courses in a community college setting [19]. Regardless of where pre-pharmacy courses are taken, students need to be transitioned from thinking about passing exams and a subordinate approach to thinking about themselves as health care providers. This journey encompasses professionalism, relational and superordinate thinking, and goal setting. Thus, PRIME incorporated these topics and, as evident from the students’ survey responses to questions 12–15, there was improvement in students’ metacognition and self-awareness of a pharmacist’s role and identity following PRIME. 

While curricular approaches have also been adopted to effectuate an understanding of what it is that pharmacists do, such as an early introduction to pharmacy subjects and topics [7], this is compounded by the fact that in the United States, there is diversity in the admissions landscape in the context of the required completion of prerequisite content for students entering into PharmD programs and the degree required to enter pharmacy school. For example, while the majority of students complete a Bachelor of Science degree or higher education including a Master of Science degree, a select few may complete required prerequisite courses from community colleges, most of which offer an Associate’s degree, and a Bachelor’s degree is not an absolute requirement for many pharmacy school admissions [19]. Thus, incoming first-professional-year cohorts may be diverse in terms of overall readiness of the students, heterogeneity of their age, and the corresponding development of the professional attitude and aptitude needed for successful progression in a rigorous health professions education program. 

Additionally, whether students arrive at the PharmD program following a formal education through the undergraduate curriculum or otherwise, in most cases they have no a priori knowledge, skills, or behaviors to encourage them to think and conduct themselves as a health care professional, most having never interacted with patients or other healthcare providers. Thus, there exists a gap in knowledge, program readiness, attitude, aptitude, and skills of starting first-year pharmacy students, many of whom need assistance in transitioning from an undergraduate student persona to that of an evolving health care professional [20]. This challenge may be programmatically addressed by implementing novel curricula that introduce incoming pharmacy students to these health professions’ attitudes, aptitudes, accountability, interdisciplinary and interprofessional awareness, and content readiness, a state-of-the-mind that may be collectively referred to as pharmacy student’s professional identity. 

Our pre-matriculation PRIME program was created to expose pre-matriculated students about to embark on their pharmacy school journey to different aspects of routine work that pharmacists are expected to perform in a variety of settings. By introducing pre-pharmacy students to different components of a pharmacist’s professional identity including the integration of foundational science knowledge in pharmacy practice, our ultimate goal was enhancing culture change and learner accountability. For example, we intentionally embedded a “bridge what you learned to pharmacy” application exercise at the end of each new concept introduced in every session (Table 1). Meetings were held with each of the faculty leading the different modules, introducing concepts related to PIF or pre-requisite content to ensure that this bridging activity was designed homogenously. Typically, the bridging activity presented real-life scenarios, simplified patient cases, “wrong scenarios” with deliberate errors, pre-recorded video snippets, or content-based problem cases. In the second week of the PRIME program, the bridging applications contained elements not only of the new concepts but also of the previous week’s work. 

### 4.2. PRIME, Professional Identity Formation, and Professionalism 

There is an urgent need to examine the relationship between professionalism and PIF and whether a relationship exists between the two terms in the context of pharmacy learners. While PIF is a way of being and is defined as the collective skills, attitudes, and behaviors associated with professionals’ engaged area of work, PIF is an umbrella term that encapsulates a plethora of developmental and behavioral components including professionalism. While PIF and professionalism are being examined in pharmacy education, there is a need to develop strategies to longitudinally incorporate both in pharmacy curricula. Additionally, working examples from pharmacy schools and colleges of the “how-to” implement and assess professionalism and PIF of learners, particularly in the P1 and P2 formative years are urgently needed. Programs such as PRIME offer an extra-curricular mechanism to introduce these concepts from the very beginning of pharmacy students’ academic careers. Furthermore, all students need to licensed as pharmacy interns at the start of the PharmD program, which will further reinforce pharmacists’ identity. 

### 4.3. Resources Needed When Considering the Transferability of PRIME to Other Pharmacy Programs 

Effort in terms of faculty hours ranged from a minimum of 3 hours to more than 14 hours for the program director in terms of classroom time, not counting time for preparation and content development. Three staff helped support the program by registering students, making flyers and brochures, sending out and monitoring student correspondence, and providing other administrative support. Faculty and staff time was covered by support from the Office of the Dean. 

### 4.4. PRIME and Pre-Matriculation Health Professions Education Programs 

Finally, PRIME significantly differed from pre-matriculation programs in pharmacy and medical education because its primary focus was to inculcate a sense of pharmacists’ professional identity in early learners, rather than solely to recapitulate and review foundational sciences content. Pre-matriculation programs in medical and pharmacy education mostly focus on preparing students for class-and program readiness. The few studies we could find in the literature mentioning interdisciplinary programs tended to concentrate on content specificity for a science-based subject matter and used test readiness and performance as major indicators of achievement [12,13,14,15]. 

### 4.5. Similarities and Differences between the Virtual and F2F Versions of PRIME

Virtual and F2F PRIME were similar in many ways including (1) mechanisms for recruiting students, (2) intent and approach, (3) inclusion of content related to professional identity formation, professionalism, prerequisite foundational sciences, pharmacy careers, time and stress management, (4) overall curricular design, especially pre-PRIME and post-PRIME proctored tests, pretest and posttest self-assessments, post-lecture “bridge to pharmacy activity”, and (5) faculty and staff time. The two versions differed from each other primarily in virtual or in-person delivery. The topic of graduate writing was the one subject added to the virtual version that the F2F version lacked. 

### 4.6. Limitations

Although we included both content-based formative quizzes and end-of-activity application exercises and used student perception surveys to gauge learners’ self-perceived growth, PRIME would have benefitted from a long-term correlational analysis with learners’ attitudes, perceptions, behaviors, and performance in the field in Introductory Pharmacy Practice Education (IPPE) clinical rotations. However, in the absence of a standardized national rubric or largely accepted consensus methods for measuring mid-to-long term professional identity formation, we conducted our analysis using methods currently available to us. In the future, we are planning to repeat PRIME in the summer after the end of the first year and before the IPPE rotations and re-conduct these surveys and tests, followed by immediate and a longer-term correlational analysis of learners’ growth in PIF. 

Another study limitation was the lack of independent perception survey validation. Although we premised our survey on the published and validated survey by Mylrea et al. [16], we added some questions to make the survey relevant to pharmacy education and pharmacists, which would benefit from a validation study in the future. 

Due to the compressed nature of PRIME, during which more than 14 topics were introduced within 48 teaching hours, it is possible that incoming students did not fully grasp some of the new concepts or content. To overcome this challenge, the entire content of PRIME is available to the students on Canvas for the first year of their study in the program, should they need to refer to it. The College is also planning to consider expanding the program, though having a commitment longer than two weeks from pre-matriculated students may pose new challenges. 

Finally, roughly 70% of the incoming cohorts in 2019 and 2020 comprised learners who self-identified as Asians. While California is diverse, this reflects the demographic of the Sacramento Metro Region and the larger Bay area surrounding the San Francisco Metro region, where our college is situated, though we acknowledge that some data or performance characteristics may be skewed due to this factor. 

## 5. Conclusions

Engaging pre-pharmacy students through carefully designed, holistic, and inclusive pre-matriculation programs offers an innovative strategy to “level the playing field” by bringing all incoming students to a similar level of understanding, appreciation, and expectation of the rigors of pharmacy school and education. While PRIME was mainly designed as a program to introduce students who were starting on their academic and professional journey to concepts related to pharmacists’ roles and responsibilities, it also offers an interesting mechanism for improving retention rates by possibly improving readiness and student engagement on many levels.

## Figures and Tables

**Table 1:** Topics included in the “Professional Identity and Me” (PRIME) pre-matriculation program.

WEEK ONE
**Day 1: Welcome and Intro to the Profession of Pharmacy in the U.S.A.**
An introduction to PRIME 2020: goals and objectives
Introduction to the profession of pharmacy in the U.S.A.: a brief overview
Biochemistry pre-test (self-assessment)
Biochemistry review lectures
Lunch
Biochemistry review lectures
“Bridge what you learned to pharmacy” activity – Biochemistry
Biochemistry post-test
*Let’s socialize!* Online bingo night and team building event
**Day 2: Pharmacists’ and Pharmacy Students’ Professional Identity**
An introduction to graduate writing: *essential skills for a pharmacist*
Professional identity: what it is and how you are different from an undergraduate
Effective teamwork strategies: *essential skills for a pharmacist*
Interprofessional education—Pharmacists’ integration in healthcare: *essential skills for a pharmacist*
Lunch
Introduction to “Top 200 Drugs”: *essential skills for a pharmacist*
Career planning for pharmacy
College student organizations and committee service opportunities
**Day 3: Professional Identity and Mental Health and Wellbeing**
Mental, physical, and emotional health in graduate programs: *essential skills for a pharmacist*
Organic chemistry pre-test (self-assessment)
Organic and medicinal chemistry review lectures
Lunch
Organic and medicinal chemistry review lectures
“Bridge what you learned to pharmacy” activity—Organic and Medicinal Chemistry
Medicinal chemistry post-test
*Let’s socialize*! Team building activity
**Day 4: Peer Support—The Professional Identity of a Pharmacy Student**
Strategies for student success in pharmacy school: *A student-led, peer-to-peer discussion*
Time and stress management panel: *student peer panel* | *essential skills for a pharmacist*
Pharmacology pre-test (self-assessment)
The fundamental principles of pharmacology: linkage with biochemistry and medicinal chemistry lectures
Lunch
Pharmacology lectures
“Bridge what you learned to pharmacy” activity and post-test—Pharmacology
*Let’s socialize! Movie night*
**WEEK TWO**
**Day 5: The Practice Mindset—Clinical and Patient-Oriented Identity of a Pharmacy Student**
A detailed discussion of pharmacy practice and clinical training schedule in the PharmD program: *essential skills for a pharmacist*
Introduction to postgraduate careers: residency and the Pharma Industry
Pre-test and review of the Nervous System
“Bridge what you learned to pharmacy” activity and post-test—Nervous System
**Day 6: Professional Identity and Professionalism—Differences and Similarities in the Work Place and Pharmacy School**
Pre-test (self-assessment) Professionalism
Professionalism in pharmacy education and the pharmacy profession: *essential skills for a pharmacist*
Lunch
Pre-test and review of the Cardiovascular System
“Bridge what you learned to pharmacy” activity and post-test—Cardiovascular System
**Day 7: Professional Identity of a Community Pharmacist—Calculations**
Calculations, percentages, and ratios: *essential skills for a pharmacist*
Lunch
Part I: An introduction to Biopharmaceutics and Pharmacokinetics
Part II: Biopharmaceutics and Pharmacokinetics—general concepts
“Bridge what you learned to pharmacy” activity and post-test—Biopharmaceutics and pharmacokinetics
**Day 8: Professional Identity of a Community Pharmacist—Calculations**
Pre-test dimensional analysis
Dimensional analysis lectures
**Professional Identity: Pharmacy Management and Operations**
Introduction to pharmacy management and operations
“Bridge what you learned to pharmacy” activity and post-test—management and operations
*Let’s Celebrate: Conclusion Ceremony—Lunch and Awards! Meet the Faculty!*

**Table 2:** **Survey questions.** The student perceptions survey instrument comprised 23 questions. The questions were developed to correlate with six summary categories related to pharmacy students’ professional identity formation such that learners’ self-perceived entry-level professional identity could emerge. These categories included (1) social and cultural awareness, (2) interprofessional awareness, (3) innovation, pharmacy careers knowledge and entrepreneurship, (4) mental health and wellness, (5) educator and public health, and (6) commitment to pharmacy and professional advocacy.

Question Number	Question Text (Answer Options for All Questions; ‘Strongly Agree’, ‘Agree’, ‘Neutral’, ‘Disagree’, ‘Strongly Disagree’)
1	It is important for me to graduate from pharmacy school.
2	PRIME made me more aware of what a profession in pharmacy entails.
3	PRIME made me excited about the pharmacy program.
4	I gained a better understanding of the subjects I will study in the pharmacy program by attending PRIME.
5	I liked the exposure I got to studying pharmacy-related subject material as an introduction to pharmacy school.
6	I think that PRIME improved my chances of performing in pharmacy school and graduating from the pharmacy program in the scheduled four years.
7	PRIME increased my interest in learning about different career choices in pharmacy.
8	PRIME increased my interest in learning about pharmacy residencies and fellowships.
9	PRIME made me feel welcomed by CNUCOP
10	PRIME made me better understand the structure of the pharmacy program.
11	I liked meeting some of the faculty and students in the pharmacy program during the PRIME.
12	PRIME identified areas where I may need more preparation.
13	PRIME helped me review material that will better my understanding of the content of the upcoming classes.
14	PRIME helped me to think about my study habits
15	During or after attending PRIME, I have created or thought about creating a timetable to manage studying.
16	PRIME afforded an opportunity to meet and interact with other incoming P1 students.
17	PRIME helped create an environment of team building.
18	PRIME helped me form alliances that will enable team studying in the future.
19	PRIME helped me learn about professional behavior in the profession of pharmacy.
20	I feel that attending PRIME gave me an advantage to perform better in pharmacy school.
21	PRIME helped me identify opportunities to improve my time management skills.
22	Attending PRIME enhanced my awareness of my knowledge base in the fundamental sciences.
23	After attending PRIME, I will be proactive in seeking academic support resources, if needed.

**Table 3:** **Comparative analysis of the F2F PRIME 2019 and Virtual PRIME 2020 cohorts.** Demographic characteristics of the two cohorts were compared concerning age in years, gender, race, and ethnic self-identification. Also included are the pre-matriculation, undergraduate average, science, and math GPAs of the incoming pharmacy learners.

Characteristic	F2F PRIME 2019 N = 58	Virtual PRIME 2020 N = 46	*p*
**Age (years)** Mean ± s.d.	24.0 ± 3.1	23.5 ± 2.3	0.42
**Gender, N (%)**			
Female	44 (75.9)	26 (56.5)	0.04 *
Male	14 (24.1)	20 (43.5)	
**Race and Ethnicity, N (%)**			
Asian	44 (75.9)	30 (65.2)	0.23
Black	1 (1.7)	2 (4.3)	0.43
Hispanic	4 (6.9)	3 (6.5)	0.94
White	8 (13.8)	7 (15.2)	0.84
Other	1 (1.7)	4 (8.7%)	0.10
**Undergraduate, Pre-matriculation GPA, Mean ± s.d.**			
Overall GPA	3.0 ± 0.4	2.9 ± 0.3	0.23
Science GPA	2.7 ± 0.5	2.6 ± 0.4	0.37
Math GPA	3.0 ± 0.6	2.7 ± 0.6	0.03*

* Statistically significant

## Data Availability

Not applicable.
